# Assessment of subclinical cardiac dysfunction by speckle-tracking echocardiography among people living with human immunodeficiency virus

**DOI:** 10.3389/fcvm.2023.1200418

**Published:** 2023-05-23

**Authors:** Chia-Te Liao, Han Siong Toh, Wei-Ting Chang, Chun-Ting Yang, Zhih-Cherng Chen, Hung-Jen Tang, Carol Strong

**Affiliations:** ^1^ Department of Public Health, College of Medicine, National Cheng Kung University, Tainan, Taiwan; ^2^ Division of Cardiology, Department of Internal Medicine, Chi Mei Medical Center, Tainan, Taiwan; ^3^ Studies Coordinating Centre, Research Unit Hypertension and Cardiovascular Epidemiology, KU Leuven Department of Cardiovascular Sciences, University of Leuven, Leuven, Belgium; ^4^ Department of Intensive Care Medicine, Chi Mei Medical Center, Tainan, Taiwan; ^5^ Institute of Clinical Medicine, College of Medicine, National Cheng Kung University, Tainan, Taiwan; ^6^ Department of Health and Nutrition, Chia Nan University of Pharmacy & Science, Tainan, Taiwan; ^7^ Institute of Clinical Pharmacy and Pharmaceutical Sciences, College of Medicine, National Cheng Kung University, Tainan, Taiwan; ^8^ Department of Internal Medicine, Chi Mei Medical Center, Tainan, Taiwan

**Keywords:** HIV/AIDS, cardiovascular disease, speckle-tracking echocardiogram, myocardial strain, subclinical myocardial dysfunction

## Abstract

**Background:**

People living with HIV (PLWH) have an increased risk of developing cardiovascular diseases (CVD). As speckle-tracking echocardiography (STE) has been used to detect subclinical myocardial abnormalities, this study aims to detect early cardiac impairment among Asian PLWH using STE and to investigate the associated risk factors.

**Methods:**

We consecutively recruited asymptomatic PLWH without previous CVD from a medical center of Taiwan, and their cardiac function was evaluated by conventional echocardiogram and STE. Enrolled PLWH were classified as antiretroviral therapy (ART)-experienced and ART-naive, and multivariable regressions were used to assess the association between myocardial strain and risk factors including traditional CVD and HIV-associated factors.

**Results:**

A total of 181 PLWH (mean age: 36.4 ± 11.4 years, 173 males) were recruited and conventional echocardiogram parameters were within normal ranges. Decreased myocardial strain across the myocardium was found, with a mean left ventricular (LV) global longitudinal strain of −18.7 ± 2.9%. The LV strain in the ART-experienced group (−19.0 ± 2.9%) was significantly better than the ART-naive group (−17.9 ± 2.8%), despite a younger age and lesser CVD risk factors in the ART-naive group. Hypertension [B = 1.92, 95% confidence interval (95% CI) 0.19–3.62, *p *= 0.029] and ART-naive with both low and high viral loads (VL) (B = 1.09, 95% CI 0.03–2.16, *p *= 0.047; and B = 2.00, 95% CI, 0.22–3.79, *p *= 0.029) were significantly associated with reduced myocardial strain.

**Conclusion:**

This is the first and largest cohort using STE to investigate myocardial strain in Asian PLWH. Our results suggest that hypertension and detectable VL are associated with impaired myocardial strain. Thus, timely ART administration with VL suppression and hypertension control are crucial in preventing CVD when making the management parallel with the improved life expectancy of PLWH on ART.

## Introduction

In the last decade, with the advancement of antiretroviral therapy (ART), increased access to care for people living with human immunodeficiency virus (PLWH), and the introduction of preventive strategies such as pre-exposure prophylaxis, there has been significant progress in the control of HIV, resulting in a decline in new HIV infections of 32% and a decline in AIDS-related deaths of 52% since 2010 globally (1). With the increasing life expectancy of PLWH, the new challenge in the burden to the healthcare systems brought by HIV/AIDS has shifted to noncommunicable diseases, particularly cardiovascular diseases (CVD).

PLWH have been shown to have an increased risk for CVD, including acute myocardial infarction, ischemic stroke, and heart failure, which yielded as one of the most common causes of non-AIDS-related deaths among PLWH on ART (2–6). In addition to traditional CVD risk factors, HIV-related factors such as low CD4 cell count and high HIV viral loads can contribute to excess CVD risks in PLHIV (2). In addition, ART's side effects and toxicities may also increase the risk for metabolic syndrome, further increasing their CVD risk (2, 7, 8). For example, some ARTs have been reported to be associated with mitochondrial toxicity or cardiotoxicity, which may increase the risk of developing cardiac dysfunction (2). Due to the lack of appropriate screening, health education, and management protocols for cardiovascular risk modifications, PLWH may not receive timely diagnosis and optimal treatments to prevent advanced cardiovascular events (9). Therefore, early recognition of cardiac impairment in CVD management becomes crucial in parallel with the improved life expectancy of the HIV-infected population in the ART era (9–11).

Echocardiogram is a commonly used method to evaluate heart function with the advantages of safety, rapidness, and noninvasiveness (10). However, the conventional echocardiogram may not be sensitive enough to detect subtle regional myocardial abnormality and may, therefore, lead to delays in CVD preventive management. Speckle-tracking echocardiography (STE), a newer quantitative ultrasound technique, can detect early myocardial abnormality by measuring myocardial strain. It has been used to assess subclinical cardiac involvement and impairment in many diseases, such as CVD-free hypertensive and diabetic patients (12, 13). Previous STE studies showed reduced myocardial strains in PLWH compared to general populations, even given normal conventional echocardiography measurements. These findings provide supporting data on STE's capability in detecting subclinical myocardial abnormality in PLWH (14–19). Nevertheless, the small sample size and limited study population may influence the robustness of evidence to generalize the application of STE.

Since most studies mainly focused on comparing myocardial strain between people with and without HIV, the association between HIV-specific factors (such as CD4 cell counts, viral loads, and ART classes) and myocardial strains is underreported and inconsistent among these studies (16, 18). Due to these gaps and limitations, this study aimed to assess the effect of STE on detecting subclinical myocardial impairment and investigate the determinants associated with early cardiac dysfunction among Asian PLWH.

## Methods

### Study cohort and participants

This retrospective study was performed in an HIV-designated tertiary medical center in southern Taiwan. In July 2017, the center employed a “Double V” program carrying out multidisciplinary healthcare for CVD in PLWH. The program provided multifaceted longitudinal healthcare, and STE was employed regularly to detect early myocardial deformity. All who consented to participate were consecutively enrolled and followed up every 6 months. In this study, the participants were identified from July 2017 to January 2020 in the “Double V” cohort. The inclusion criteria were asymptomatic adults with HIV infection (aged between 20 and 65 years) who consented to participate in the study. The exclusion criteria comprised those with existing CVDs, and detailed exclusion criteria are listed in the Supplementary material. The selected subjects were divided into two subgroups, ART-experienced and ART-naive PLWH, to examine the influence of ART treatment on the myocardial strain. For not delaying the initiation of ART, those who received baseline cardiac STE before or within 2 months after starting ART are classified as ART-naive. PLWH's clinical and laboratory characteristics at the enrollment were collected from electrical medical records. This study was approved by the Institutional Review Board of Chi Mei Medical Center (10605-003).

### Conventional and speckle-tracking echocardiographic measurements

The echocardiography examination was performed by two trained cardiologists (C-TL and W-TC) using a 3.5 MHz multiphase-array probe of the GE Vivid E9 system (GE-Vingmed Ultrasound AS, Horten, Norway) with a standardized protocol (20). The echocardiogram operation and measurement were based on the recommendations of the American Society of Echocardiography guidelines (20) (see the Supplementary material). The speckle-tracking strain measurements were performed offline using computer software (EchoPAC; GE-Vingmed Ultrasound AS) and were obtained by calculating the percentage of myocardium deformation. The peak systolic longitudinal strain was acquired from the three standard apical views (apical 4-, 3-, and 2-chamber views). Left ventricular (LV) global longitudinal strain (GLS) and right ventricle (RV) septal and free-wall longitudinal strain were estimated using the mean peak systolic longitudinal strain (%) (Supplementary Figure S1). Since the speckles over the endocardium were tracked during myocardial systole, where the endocardium in the longitudinal direction became thinner, the values were presented as negative percentages for the longitudinal strain. Two image readers measured the parameters independently and were blinded to the study population's clinical characteristics and laboratory data. The intraobserver and interobserver variabilities were validated in our previous study (21).

### Variables and echocardiographic parameters

Demographic characteristics, including age, sex, body mass index (BMI), waistline, comorbidities, family history, history of AIDS, and ART prescription, were collected. Laboratory data comprised biochemistry, lipid profiles, fasting serum glucose, D-dimer, high-sensitivity C-reactive protein (hsCRP), CD4 T-cell counts, and HIV viral load. HIV viral load was classified as high if more than 10^5^ copies/mm^3^. ART regimens were categorized according to the nucleoside reverse-transcriptase inhibitors (NRTI) backbone and the third agents, including non-nucleoside reverse-transcriptase inhibitors (NNRTI), protease inhibitors (PI), and integrase strand transfer inhibitors (INSTI). Different NRTIs were analyzed separately to investigate the impact of individual regimens, including tenofovir disoproxil fumarate (TDF), tenofovir alafenamide, abacavir. and zidovudine. Regimen with a booster such as ritonavir or cobicistat was also analyzed as an individual variable. Conventional echocardiographic parameters comprised left atrial and ventricular sizes, E/A, E/e’, RV S’, RV systolic pressure (RVSP), and LV ejection fraction (LVEF). Myocardial strain data included LVGLS at the different myocardial layers (endo-, mid-, and epi-myocardium) and RV longitudinal strain at the septum and free wall. Detailed segmental strain data were also collected to evaluate the individual regional function and represented with the bull's eye map.

### Statistical analysis

Continuous variables were reported as a mean ± SD or median with interquartile range (IQR) if the variables were not normally distributed. Categorical variables were presented as number and percentage. A simple independent *t* or Mann–Whitney *U* test was used to compare the continuous variables between the two subgroups. Categorical variables were tested by the chi-square or Fisher exact tests. Pearson's or Spearman's analyses were performed to examine the correlation between myocardial strain and the baseline variables. Regarding the variables with a *p*-value of <0.1 in the univariate analysis, the multivariate regression model was used to estimate the association. In the subgroup analysis, an Eta Coefficient test was carried out to determine the strength of the association between myocardial strain and different ARTs in the experienced group, and the effect size was interpreted as small (0.01), moderate (0.06), and large (0.14) according to the eta-square value (22). A *p*-value smaller than 0.05 was considered statistically significant, and all statistical analyses were performed using SPSS 28 software.

## Results

### Baseline characteristics

A total of 181 PLWH (age 36.4 ± 11.4 years, 173 males) were enrolled in this study, including 133 ART-experienced (age 38.5 ± 11.5 years) and 48 ART-naive PLWH (age 30.4 ± 8.6 years) (Table 1). Regarding traditional risks for CVD, the percentages of current smoker and having family history of CVD were 35.9% and 30.9%, respectively. ART-experienced PLWH had statistically higher percentages of hypertension (9%), dyslipidemia (15%), and a family history of CVD (37.6%) compared with ART-naive PLWH. In addition, ART-experienced PLWH also have a higher BMI and waist circumference. The laboratory data were within normal ranges, including lipid profiles, kidney and liver function, uric acid, D-dimer, and hsCRP (Table 1). Notably, total cholesterol was higher in ART-experienced PLWH, whereas ART-naive PLWH had a higher estimated glomerular filtration rate and D-dimer.

**Table 1:** The basic characteristics of people living with HIV in this study.

	Total PLWH (*N* = 181)	ART-experienced PLWH (*N* = 133)	ART-naive PLWH (*N* = 48)
Age, mean ± SD	36.4 ± 11.4	38.5 ± 11.5	30.4 ± 8.6
Gender (male:female)	173:8	125:8	48:0
Body mass index (kg/m^2^), mean ± SD	22.4 ± 3.5	22.8 ± 3.5	21.3 ± 3.0
Waist circumference (cm)	82 ± 10.4	84.1 ± 10.3	76.1 ± 8.4
Traditional risk factors of CVD			
Hypertension, *n* (%)	12 (6.6)	12 (9.0)	0 (0)
Diabetes, *n* (%)	5 (2.8)	5 (3.8)	0 (0)
Dyslipidemia, *n* (%)	20 (11.0)	20 (15.0)	0 (0)
Current smoker, *n* (%)	65 (35.9)	46 (34.6)	19 (39.6)
Ex-smoker, *n* (%)	15 (8.3)	12 (9.0)	3 (6.3)
Family history of CVD, *n* (%)	56 (30.9)	50 (37.6)	6 (12.5)
Laboratory data			
Total cholesterol (mg/dl)	169.7 ± 36.2	173.7 ± 37.1	158.9 ± 31.2
HDL cholesterol (mg/dl)	42.1 ± 10.4	43.0 ± 10.2	39.7 ± 10.5
LDL cholesterol (mg/dl)	104.5 ± 29.3	106.8 ± 30.3	98.1 ± 25.4
Triglyceride (mg/dl)	134.8 ± 89.7	139.7 ± 96.1	121.2 ± 68.1
Glucose (mg/dl)	94.3 ± 14.8	95.4 ± 16.5	91.3 ± 7.4
HbA1c (%)	5.5 ± 0.5	5.6 ± 0.5	5.5 ± 0.5
GOT (U/L)	26.4 ± 20.2	26.7 ± 22.5	25.6 ± 11.8
Uric acid (mg/dl)	6.3 ± 1.3	6.2 ± 1.3	6.5 ± 1.2
eGFR (ml/min/1.73 m^2^)	94.1 ± 19.8	91.4 ± 19.0	101.9 ± 20.2
Albumin–creatinine ratio, median (IQR)	7.3 (5.2–12.1)	8.0 (5.8–12.2)	5.6 (3.8–9.3)
D-dimer (mg/L), median (IQR)	194.5 (129.0–288.7)	172.9 (125.1–252.1)	274.3 (156.4–376.0)
hsCRP (mg/dl), median (IQR)	1.2 (0.5–2.6)	1.2 (0.5–2.3)	0.9 (0.6–1.75)
**HIV-relevant data**			
Years of HIV diagnosis, median (IQR)	3 (0–6)	4 (2–6)	0 (0–0)
Months on ART, median (IQR)	29 (2–53)	42 (24–69)	0 (0–0)
CD4 count (per mm^3^), mean ± SD	485.5 ± 249.6	538.8 ± 244.4	346.3 ± 209.6
CD4 count < 200/mm^3^, *n* (%)	20 (11.0)	9 (6.8)	11 (22.9)
Viral load > 10^5^ copies/mm^3^, *n* (%)	11 (6.1)	0	11 (22.9)
History of AIDS, *n* (%)	74 (40.9)	60 (45.1)	14 (29.2)
Recreational drug used, *n* (%)	18 (9.9)	13 (9.8)	5 (10.0)
Hepatitis B coinfection, *n* (%)	18 (9.9)	16 (12.0)	2 (4.2)
Hepatitis C coinfection, *n* (%)	11 (6.1)	10 (7.5)	1 (2.1)
**ART types**			
NRTI + INSTI, *n* (%)	79 (43.6)	33 (24.8)	46 (95.8)
NRTI + PI, *n* (%)	50 (27.6)	50 (37.6)	0
NRTI + NNRTI, *n* (%)	51 (28.2)	49 (36.8)	2 (4.2)
INSTI + PI, *n* (%)	1 (0.6)	1 (0.8)	0
NRTI with TDF	71 (39.2)	69 (51.9)	2 (4.2)
NRTI with TAF	32 (17.7)	12 (9.0)	20 (41.7)
NRTI with ABC	54 (29.8)	28 (21.1)	26 (54.2)
NRTI with AZT	23 (12.7)	23 (17.3)	0
Regimen with booster, *n* (%)	61 (33.7)	44 (33.1)	17 (35.4)

PLWH, people living with HIV; SD, standard deviation; CDV, cardiovascular diseases; HDL, high-density lipoprotein; LDL, low-density lipoprotein; HbA1c, hemoglobin A1c; GOT, glutamic oxaloacetic transaminase; eGFR, estimated glomerular filtration rate; hsCRP, high-sensitivity C-reactive protein; ART, antiretroviral therapy; AIDS, acquired immunodeficiency syndrome; NRTI, nucleoside reverse-transcriptase inhibitors; NNRTI, non-nucleoside reverse-transcriptase inhibitors; PI, protease inhibitor; INSTI, integrase strand transfer inhibitor; TDF, tenofovir disoproxil fumarate; TAF, tenofovir alafenamide; ABC, abacavir; AZT, zidovudine; IQR, interquartile range.

Regarding HIV-relevant data, 40.9% of the total PLWH had a history of AIDS, 9.9% reported using recreational drugs, 9.9% had hepatitis B, and 6.1% had hepatitis C coinfection. The medians of years of HIV diagnosis and months of ART use in the ART-experienced PLWH were 4 years (IQR, 2–6 years) and 42 months (IQR, 24–69 months). Reasonably, mean CD4 T-cell counts in the ART-experienced group were higher than in the ART-naive group. The percentages of those with CD4 less than 200/mm^3^ and HIV viral loads more than 10^5^ copies/mm^3^ in the ART-naive group (22.9% and 22.9%) were higher than the ART-experienced group (6.8% and 0%). NRTI + INSTI, NRTI + PI, and NRTI + NNRTI each constituted approximately one-third of ART prescriptions among the ART-experienced PLWH, while the ART-naive PLWH mostly started with an INSTI-based regimen. TDF constituted the most prescribed NRTI in the ART-experienced group, but abacavir was the predominantly used backbone in the ART-naive group.

### Echocardiographic measurement

**Figure 1:**
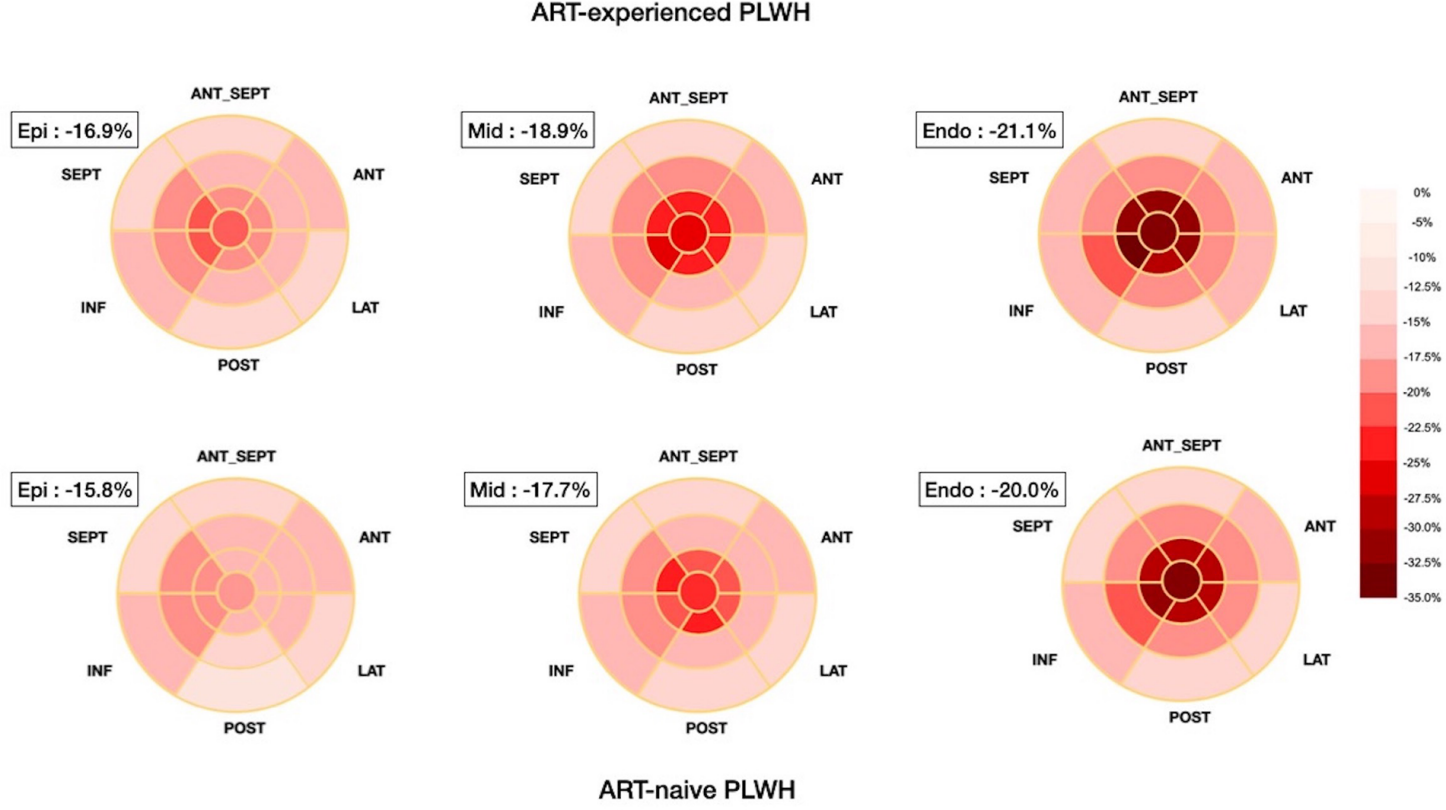
Simplified bull's eye maps present the segmental myocardial strain in different layers in ART-experienced and ART-naive people living with HIV. The more negative strain values marked with darker red color mean better myocardial function. The strain decreases from the endocardium to the epicardium, and the value in the ART-naive group is lower than the ART-experienced group across the three layers consistently. ART, antiretroviral therapy.

The echocardiographic data and the bull's eye maps representing segmental myocardial strains are listed in Table 2, Figure 1, and Supplementary Table S1. ART-experienced PLWH had greater values of left atrial chamber diameter and LV end-diastolic diameter, whereas ART-naive PLWH had a greater E/A ratio. Although their LVEF and diastolic function are within the normal range according to conventional echocardiography, 10.5% of PLWH have RVSP of more than 25 mmHg.

**Table 2:** Echocardiography data among people living with HIV in this study.

	Total PLWH (*N* = 181)	ART-experienced (*N* = 133)	ART-naive (*N* = 48)	*p*-value
Conventional echocardiography				
LA diameter (mm)	30.1 ± 5.1	30.6 ± 5.3	28.5 ± 4.3	0.013
LVESD (mm)	29.5 ± 4.3	29.8 ± 4.6	28.9 ± 3.1	0.251
LVEDD (mm)	47.7 ± 5.9	48.3 ± 6.0	46.1 ± 5.4	0.027
LVMI (g/m^2^)	66.8 ± 16.6	67.6 ± 16.0	64.6 ± 18.3	0.297
LVEF (%)	66.0 ± 5.5	66.2 ± 5.6	65.2 ± 5.3	0.296
E/A	1.3 ± 0.4	1.3 ± 0.4	1.5 ± 0.5	<0.001
E/e’	6.0 ± 2.0	6.2 ± 1.8	5.7 ± 2.7	0.201
S’ (cm/s)	14.8 ± 2.1	14.8 ± 2.2	14.9 ± 1.7	0.878
RVSP (mmHg)	16.9 ± 6.6	17.0 ± 6.9	16.6 ± 5.6	0.700
RVSP > 25 mmHg (*n*, %)	19 (10.5%)	15 (11.3%)	4 (8.3%)	0.781
Speckle-tracking echocardiography				
LVGLS (%)	−18.7 ± 2.9	−19.0 ± 2.9	−17.9 ± 2.8	0.023
LV LS at endo-myocardium (%)	−20.8 ± 3.2	−21.1 ± 3.2	−20.0 ± 3.1	0.039
LV LS at mid-myocardium (%)	−18.6 ± 2.9	−18.9 ± 2.9	−17.7 ± 2.8	0.022
LV LS at epi-myocardium (%)	−16.6 ± 2.7	−16.9 ± 2.7	−15.8 ± 2.6	0.015
RV septal LS (%)	−17.8 ± 3.7	−17.5 ± 3.7	−18.6 ± 3.8	0.079
RV free-wall LS (%)	−21.0 ± 6.4	−20.9 ± 6.9	−21.1 ± 5.0	0.874

The *p*-value of < 0.05 presents the statistically significant difference between the ART-experienced and ART-naive groups. ART, anti-retroviral therapy; LA, left atrium, LVEDD; left ventricular internal diameter end diastole; LVESD, left ventricular internal diameter end systole; LVMI, left ventricle mass index; RVSP, right ventricular systolic pressure; LVEF, left ventricular ejection fraction; LVGLS, left ventricular global longitudinal strain; LS, longitudinal strain; RV, right ventricular.

For myocardial strain, the PLWH's LVGLS was −18.7 ± 2.9%, and the strain decreased from the endocardium to the epicardium (Table 2 and Figure 1). Compared with ART-experienced PLWH, ART-naive PLWH had significantly reduced strain values in each layer, i.e., −22.1 ± 3.2% vs. −21.0 ± 3.6% in the endo-myocardium (*p *= 0.039), −18.9 ± 2.9% vs. −17.7 ± 2.8% in the mid-myocardium (*p *= 0.022), and −16.9 ± 2.7% vs. −15.8 ± 2.6% in the epi-myocardium (*p *= 0.015). Notably, despite the significant difference in layer-specific strain, the difference in LV segmental myocardial strains between the two subgroups was insignificant (Supplementary Table S1). The RV septal and free-wall longitudinal strains in PLWH were −17.8 ± 3.7% and −21.0 ± 6.4%, respectively. Both ART groups did not have significantly different RV septal and free-wall longitudinal strains.

### Factors associated with the LVGLS

After multivariable adjustment, hypertension was associated with poorer LVGLS [B = 1.902, 95% confidence interval (95% CI), 0.19–3.62, *p *= 0.029] among PLWH. Moreover, ART-naive PLWH have significantly reduced LVGLS compared to ART-experienced PLWH (B = −1.12, 95% CI −3.43 to −0.17, *p *= 0.023), despite low (<10^5^/mm^3^, B = 1.09, 95% CI 0.03–2.16, *p *= 0.047) or high viral load (≥10^5^/mm^3^; B = 2.00, 95% CI 0.22–3.79, *p *= 0.029) (Table 3). Those with high viral load have a greater effect size and, thus, are more likely to have poorer myocardial deformation. In the subgroup analysis, we found a lack of significant correlation between different ART regimens and LVGLS in ART-experienced PLWH (Supplementary Table S2). An eta-square value of 0.028 indicated a low association between myocardial strain and the types of NRTI.

**Table 3:** Association between left ventricular global longitudinal strain and individual factors among total people living with HIV (*N* = 181).

	Univariable analysis		Multivariable analysis	
	B estimate (95% CI)	*p*-value	B estimate (95% CI)	*p*-value
Gender (Male)	1.17 (−0.92 to 3.27)	0.270	—	—
Age	−0.03 (−0.06 to 0.01)	0.174	—	—
Body mass index	0.07 (−0.05 to 0.20)	0.244	—	—
Waist circumference	0.03 (−0.02 to 0.07)	0.231	—	—
Hypertension (yes vs. no)	1.55 (−0.19 to 3.26)	0.078	1.92 (0.19–3.62)	0.029
History of AIDS (yes vs. no)	0.57 (−0.31 to 1.44)	0.203	—	—
CD4 counts at enrollment	0.001 (−0.002 to 0.001)	0.713	—	—
ART-experienced vs. ART-naive groups	−1.12 (−3.43 to −0.17)	0.023	—	—
Viral loads ≥ 10^5^/mm^3^ (yes vs. no)	1.62 (−0.16 to 3.43)	0.076	—	—
Viral loads < 10^5^/mm^3^ in the ART-naive group^a^	—	—	1.09 (0.03–2.16)	0.047
Viral loads ≥ 10^5^/mm^3^ in the ART-naive group^a^	—	—	2.00 (0.22–3.79)	0.029

CI, confidence interval; AIDS, acquired immunodeficiency syndrome; ART, antiretroviral therapy.

The multivariable analysis only adjusted the variable with the ART-experience group in the univariable analysis.

^a^ Reference group: the ART-experience group.

## Discussion

This cross-sectional study is significant as it is the first and the largest cohort using STE to investigate myocardial strain in Asian PLWH population. The results show that ART-experienced PLWH have reduced LVGLS than ART-naive PLWH, despite the younger age and lower traditional CVD risk in the ART-naive group. Multiple regression analysis revealed that hypertension, ART-naive status, and high viral loads are associated with reduced LVGLS in PLWH. However, the association between the ART categories and LVGLS change was insignificant in our analysis.

The mechanism underlying HIV-associated cardiomyopathy is typically multifactorial, resulting from direct HIV toxicity, opportunity infections, autoimmunity, severe immunosuppression, or nutritional deficiencies (23). Previous studies have shown that PLWH have a reduced LVGLS even in the absence of other risk factors and comorbidities (12–19). In our analysis, PLWH's layer-specific LV strain was −20.8%, −18.6%, and −16.6% at endo-, mid-, and epi-myocardium, respectively. These values were relatively reduced compared to those in the healthy Asian subjects, i.e., −22.8%, −20.0%, and −17.5% at endo-, mid-, and epi-myocardium, respectively (24). This finding was consistent with previous studies, demonstrating the asymptomatic reduction in myocardial deformation in PLWH even though they did not have CVDs (15–19). In addition, Mirea et al. and our study have both shown that the strain values decrease from the endocardium to the epicardium (19). The differences in layer-specific strain values may be due to the distribution of fiber angles of human heart, and the pattern of strain reduction may aid in distinguishing the determinant of myocardial impairment (25). For example, hypertension, hypercholesterolemia, and drug-related cardiotoxicity (such as anthracycline) can lead to a marked strain reduction in the endo-myocardium, while transmural infarction, diabetes, and hypertrophic cardiomyopathy have consistent decreases across all layers (24). Our study found that PLWH's layer-specific strains reduced similarly in each layer of the myocardium, irrespective of being ART-experienced or ART-naive. The finding suggests that HIV toxicity *per se* instead of ART-related cardiotoxicity or hypercholesterolemia is the primary factor causing cardiac impairment in PLWH.

In recent years, ART has considerably enhanced the life expectancy of PLWH. However, the prolonged use of certain ARTs raises concerns regarding the development of CVD, owing to their potential cardiac toxicity and increased risk of dyslipidemia (11, 23). Despite these reports, our subgroup analysis did not identify a correlation between specific ART types and myocardial strain. Furthermore, a previous study by Rodrigues et al. discovered that PLWH receiving ART exhibited a marginally smaller reduction in LVGLS compared to those not receiving ART (−18.4% and −17.7% in ART and non-ART groups, respectively) (17). In our study, with a larger sample size, we detected a significant association between improved LVGLS and ART use in PLWH. Notably, despite the higher age, BMI, and prevalence of hypertension and dyslipidemia in the ART-experienced group, they still demonstrated better myocardial strain than ART-naive individuals after adjusting for viral loads and CVD risk factors. These findings underscore the importance of timely ART administration upon HIV diagnosis and suggest that the benefits to myocardial health may outweigh the potential adverse effects of ART and other contributing factors.

On the other hand, although CD4 T-cell counts were reported to be negatively correlated with the reduction of myocardial strain (16, 18), we did not observe a significant association in our study, even when considering inflammatory markers like D-dimer and hsCRP. However, it is worth noting that previous studies did not consider the intermediate effects of CD4 count and viral loads. In our study, the majority of ART-experienced PLWH had normal-range CD4 counts, and the sample size with low counts might be too small to reach the power of statistical significance. While CD4 counts can fluctuate due to factors other than HIV treatment, viral load is widely regarded as a more critical measure of HIV status in individuals undergoing long-term ART treatment (26, 27). Likewise, our study found that change in myocardial strain was associated with PLWH's viral loads rather than CD4 counts, suggesting that viral loads may be a more meaningful indicator when evaluating HIV-associated cardiomyopathy. Therefore, maintaining undetectable viral loads may play a crucial role in preventing CVD among PLWH.

Recently, a systematic review showed that PLWH are at an increased risk of developing hypertension, with those on ART having a higher prevalence due to increasing age, BMI, and chronic inflammation (28). This is particularly concerning as hypertension is among the most significant risk factors for CVD, potentially leading to cardiac impairment and reduced myocardial strain (29). In our study, we observed that the impact of hypertension on myocardial function was noticeable even in a relatively young age, highlighting the importance of hypertension management in PLWH. Regular blood pressure measurements should be performed, and antihypertensive therapy with lifestyle modifications should be initiated if the blood pressure exceeds the target of 130/80 mmHg (30). However, whether prompt management of hypertension might improve myocardial strain requires further STE study to follow up the measurements in PLWH.

In a previous study, the prevalence of RV dysfunction in PLWH was 11% with the echocardiographic definition (31). It has been observed that early RV impairment can be detected in asymptomatic PLWH with significantly reduced RV free-wall longitudinal strain (16). Our results regarding RV were consistent with this previous study. While the mechanisms of isolated RV hypertension may be similar to those of LV, pulmonary hypertension is another known risk factor for RV dysfunction, and PLWH have an increased risk of developing pulmonary hypertension (31). In our analysis, the percentage of pulmonary hypertension was relatively high compared to previous studies, with an echocardiographic definition of RVSP greater than 25 mmHg (32). We applied a broader definition of pulmonary hypertension to understand the prevalence of mild or early pulmonary hypertension change in PLWH. Further studies are necessary to investigate whether the higher RVSP affects RV strain and whether it may progress into symptomatic pulmonary hypertension.

There are some limitations in this study. First, the image quality of STE is the primary technical limitation (33). Although operators and observers were trained and qualified, the blurred endocardial border and insufficiency to accurately capture all phases of the cardiac cycle due to low temporal and spatial resolution or inadequate frame rates may influence the result readings. Research-driven updating of both hardware and software is warranted to improve the feasibility. Second, healthy subjects were not enrolled for comparison in this study. This study aimed to provide the values of PLWH's myocardial strains and assess the causes associated with subclinical myocardial abnormality. Despite lacking a healthy control group, our data were more positive than the strain values of the healthy Asian subjects and consistent with the previous findings, which may strengthen the robustness of the results. Third, the causal relationship of the data should be cautiously interpreted in the cross-sectional study. Future research on the longitudinal trajectory of myocardial strain change after ART is needed to examine their relationship. Finally, although this study had a larger sample size of PLWH and presented the feasibility of detecting subclinical myocardial impairment among Asian PLWH, the relatively small sample size of the ART-naive group may limit the conclusions that can be drawn from multivariable analyses in this subgroup, which can be attributed to fewer newly diagnosed HIV-infected individuals remaining ART-naive, i.e., the advancement of ART, increased access to care for PLWH, and the introduction of preventive strategies such as pre-exposure prophylaxis. Future studies with larger sample sizes are needed to confirm our findings.

## Conclusion

As the largest cohort investigating myocardial strain in the Asian PLWH population, our findings supported the application of STE in detecting subclinical myocardial impairment among PLWH and helped understand the relevant risk factors. Timely ART administration, viral load suppression, and hypertension control are crucial in preventing worsening myocardial function when making the management parallel with the improved life expectancy of the HIV-infected population in the ART era.

## Data Availability

The original contributions presented in the study are included in the article/**Supplementary Material**, further inquiries can be directed to the corresponding author.
