# Supplementary material for: Assessment of subclinical cardiac dysfunction by speckle-tracking echocardiography among people living with human immunodeficiency virus
Source: Front Cardiovasc Med. 2023 May 23;10:1200418. doi: 10.3389/fcvm.2023.1200418 (PMC10242012; doi:10.3389/fcvm.2023.1200418)
Supplement: Supplementary file 1 [file Table1.docx]

**Supplementary materials**

**Assessment of Subclinical Cardiac Dysfunction by Speckle Tracking Echocardiography among People Living with HIV**

Chia-Te Liao, Han Siong Toh, Wei-Ting Chang, Chun-Ting Yang, Zhih-Cherng Chen, Hung-Jen Tang, Carol Strong

**Supplementary methods: Page 2-3**

**Supplementary Table S1**: Page 4-5

Left ventricular segmental myocardial strain values.

**Supplementary Table S2: Page 6-7**

The correlation between the left ventricular global longitudinal strain and the ART-related variables.

**Supplementary methods**

*Echocardiographic measurements*

According to the American Society of Echocardiography guidelines, all 2-D echocardiography measurements included chamber size, left ventricle (LV) mass index, and LV systolic and diastolic function. (1) Left atrial (LA) size and volume, LV volume, and LV systolic function of LV ejection fraction (LVEF) were assessed using a 2-D biplane via 4-chamber and 2-chamber view on the echocardiography. The LV diastolic function assessment included trans-mitral early filling velocity (E) to atrial velocity (A) ratio and tissue Doppler imaging parameters from the apical 4-chamber view. Peak systolic annular velocity (S’) and early (e’) and late (a’) annular diastolic velocities were measured in both the ventricles. Right ventricular (RV) function was assessed by tricuspid annular plane systolic excursion, which represent the distance of the systolic excursion of the RV annular plane toward the apex. If the LV regional border cannot be well-defined, the LVEF was estimated to the nearest 5% using all available views. Given poor image quality, i.e., poor 2D or speckle-tracking image, E-A wave fusion, or missing waveforms, incomplete parameters of the measurements, these images were excluded in the outcome analyses.

*Speckle-tracking echocardiography analysis*

The study recorded standard apical 4-, 2-, and 3-chamber views in digital loops for deformation analysis of the LV. The images were obtained at frame rates of 70–90/s and store for three cycles. The speckle-tracking strain measurements were performed offline using computer software (EchoPAC; GE-Vingmed Ultrasound AS). Based on the recommendation, (45) the endocardial border was manually traced at end-systole.

The strain analysis of the interest region was adjusted manually. Given insufficient tracking, manual correction of the endocardial tracing was attempted; if still unsatisfactory, or inadequate image quality (defined as poor visualization or poor tracking of >2 ventricular segments, segment dropout, missing view, or significant foreshortening of the LV), the study was excluded from the final analysis.

The peak systolic longitudinal strain was acquired from the three standard apical views (apical 4, 3, and 2 chamber views). LV global longitudinal strain (LVGLS) was estimated using the mean peak systolic longitudinal strain from the three apical views. Longitudinal strain (%) was recorded from the apical four-chamber view.

Myocardial strain is to calculate the percentage of deformation of the myocardium. The value is measured using continuous natural acoustic speckle tracking frame by frame using the end-systolic distance between two speckles of tracked myocardium to minus the original distance between those two particles all over the original length. The values are negative percentages for the longitudinal strain because the speckles over the endocardium are tracked during myocardial systole, where the endocardium in the longitudinal and circumferential direction becomes thinner.

The image reader was blinded to the clinical characteristics of the study population and the laboratory tests. The second reader was blinded to the study group and their characteristics, and she made independent measurements. The intra-observer variability was assessed using the correlation coefficient between the individual reader’s two analyses of each echocardiogram. The inter-observer variability was examined using a reanalysis of twenty randomly selected echocardiograms (ten from each group). The reliability has been validated in the previous study. (2)

**References:**

Nagueh SF, Smiseth OA, Appleton CP, Byrd BF, Dokainish H, Edvardsen T, et al. Recommendations for the evaluation of left ventricular diastolic function by echocardiography: an update from the American Society of Echocardiography and the European Association of Cardiovascular Imaging. European Journal of Echocardiography. 2016;17(12):1321-60.

Chang WT, Feng YH, Kuo YH, Chen WY, Wu HC, Huang CT, Huang TL, Chen ZC. Layer-specific distribution of myocardial deformation from anthracycline-induced cardiotoxicity in patients with breast cancer-From bedside to bench. Int J Cardiol. 2020 Jul 15;311:64-70.

| **Supplementary Table S1.** Left ventricular segmental myocardial strain values | | | | |
| --- | --- | --- | --- | --- |
|  | **Total PLWH**  **(N=181)** | **ART-experienced PLWH**  **(N=133)** | **ART-naive PLWH**  **(N=48)** | **P value** |
| **LVGLS** | -18.7 ± 2.9 | **-19.0 ± 2.9** | **-17.9 ± 2.8** | **0.02** |
| Mid septal | -18.4 ± 3.1 | -18.5 ± 3.1 | -18.2 ± 3.2 | 0.20 |
| Mid lateral | -17.2 ± 4.3 | -17.5 ± 4.3 | -16.6 ± 4.3 | 0.67 |
| Mid inferior | -19.8 ± 3.3 | -19.9 ± 3.2 | -19.7 ± 3.5 | 0.33 |
| Mid anterior | -17.8 ± 4.2 | -17.9 ± 4.2 | -17.4 ± 4.1 | 0.47 |
| Mid posterior | -16.9 ± 4.3 | -17.2 ± 4.0 | -16.3 ± 5.0 | 0.23 |
| Mid anteroseptal | -17.6 ± 4.1 | -17.8 ± 4.0 | -16.9 ± 4.2 | 0.19 |
| Epi septal | -17.8 ± 3.0 | -17.8 ± 2.9 | -17.6 ± 3.1 | 0.63 |
| Epi lateral | -16.3 ± 4.1 | -16.6 ± 4.1 | -15.6 ± 4.2 | 0.17 |
| Epi inferior | -19.0 ± 3.2 | -19.1 ± 3.1 | -18.5 ± 3.2 | 0.20 |
| Epi anterior | -17.0 ± 3.9 | -17.2 ± 3.9 | -16.7 ± 3.9 | 0.45 |
| Epi posterior | -15.5 ± 4.3 | -15.9 ± 3.9 | -14.5 ± 5.1 | 0.06 |
| Epi anteroseptal | -16.3 ± 3.9 | -16.6 ± 3.8 | -15.7 ± 4.0 | 0.16 |
| Endo septal | -19.3 ± 3.4 | -19.4 ± 3.4 | -18.8 ± 3.4 | 0.33 |
| Endo lateral | -18.3 ± 4.7 | -18.5 ± 4.7 | -17.7 ± 4.5 | 0.26 |
| Endo inferior | -20.9 ± 3.6 | -20.8 ± 3.4 | -21.0 ± 4.0 | 0.78 |
| Endo anterior | -18.7 ± 4.3 | -18.9 ± 4.2 | -18.3 ± 4.4 | 0.43 |
| Endo posterior | -18.5 ± 4.6 | -18.6 ± 4.4 | -18.2 ± 5.1 | 0.64 |
| Endo anteroseptal | -19.1 ± 4.5 | -19.4 ± 4.4 | -18.5 ± 4.6 | 0.25 |
| PLWH, people living with HIV, LVGLS, left ventricular global longitudinal strain; | | | | |

| **Supplementary Table S2. The correlation between the left ventricular global longitudinal strain and the ART-related variables among ART-experienced people living with HIV (N=133)** | | |
| --- | --- | --- |
|  | **Left ventricular global longitudinal strain** | |
| *Correlation* | **r coefficient** | ***p*-value** |
| NRTI+INSTI | 0.02 | 0.819 |
| NRTI+PI | 0.01 | 0.948 |
| NRTI+NNRTI | -0.01 | 0.932 |
| INSTI+PI | -0.09 | 0.301 |
| ART with booster | -0.05 | 0.590 |
| *Effect size of association* | *Value of Eta square* | |
| Different NRTI types (TDF; TAF; ABC; AZT) | 0.028 | |
| AIDS, acquired immunodeficiency syndrome; ART, antiretroviral therapy; ART, anti-retroviral agents, NRTI, nucleoside reverse-transcriptase inhibitors; NNRTI, non-nucleoside reverse transcriptase inhibitors; PI, protease inhibitor; INSTI, Integrase strand transfer inhibitor; TDF, tenofovir disoproxil fumarate; TAF, tenofovir alafenamide; ABC, abacavir; AZT, zidovudine. | | |
